# Effect of nursing intervention program using abdominal palpation of Leopold’s maneuvers on maternal-fetal attachment

**DOI:** 10.1186/1742-4755-10-12

**Published:** 2013-02-19

**Authors:** Miyuki Nishikawa, Hisataka Sakakibara

**Affiliations:** 1 Department of Nursing, Kyoto Koka Women’s University, 38 Kadono-cho, Nishikyogoku, Kyoto, Ukyo-ku 615-0882,Japan; 2 Nagoya University Graduate School of Medicine, 1-1-20 Daiko-minami, 461-8673, Nagoya, Higashi-ku, Japan

**Keywords:** Pregnancy, Nonrandomized trial, Maternal–fetal attachment, Abdominal palpation, Fetal position awareness

## Abstract

**Background:**

The aim of this study was to investigate whether a nursing intervention program using abdominal palpation would improve maternal-fetal relationships of pregnant women.

**Methods:**

The subjects were Japanese women aged less than 40 years with singleton pregnancies. A nursing intervention involving abdominal palpations of Leopold’s Maneuvers was performed for the intervention group (n = 35) in the 30th, 32nd, and 34th weeks’ gestation, while ordinary health-related advice was provided to the control group (n = 73) in the corresponding period.

**Results:**

At the 30th (baseline) week, no intergroup differences were observed. However, the intervention group showed higher Prenatal Attachment Inventory (PAI) scores in the 34th (*P* < 0.01) and 36th weeks (*P* < 0.05) as well as a higher frequency of talking to the fetus in the 32nd (*P* < 0.01), 34th (*P* < 0.01), and 36th weeks (*P* < 0.05). Furthermore, Fetal position awareness score in the 32nd, the 34th, and the 36th weeks were higher in the intervention group than in the control group (*P* < 0.001).

**Conclusions:**

The present findings have suggested that nursing interventions involving abdominal palpations can develop the maternal–fetal relationship. Further random controlled trials are warranted to ascertain this observation.

## Background

Maternal–fetal attachment is defined as a mother’s affiliation with her child [1]. The development of maternal–fetal attachment greatly affects the postnatal child-care environment and child development [2-4]. It may influence mother–infant attachment after delivery and the maternal ability to care for the infant [5-7]. Hence, nursing interventions on expectant mothers are important for supporting the development of maternal–fetal attachment.

In Japan, the number of consultations for child abuse has been increasing in recent years [8], and children who are 3 years or younger account for approximately 80% of the children who die, with 0-year-old infants accounting for 60%. The main abusers of children are their mothers, and it is of primary importance that child abuse is identified at an early stage. A strong correlation has been reported between high-risk children and child abuse [9]. Higher maternal–fetal attachment would be necessary to be developed by nursing care in the pregnancy period.

There are several studies to report effective intervention programs for the development of maternal-fetal attachment in expectant mothers, such as an intervention using fetal palpation [10], offering the knowledge of the child care for couples [11], providing information and teaching stress management methods [12], expressing affection to the fetus by writing letters and talking [13], and having singing, dancing, and massage-through-the-womb sessions [14]. Meanwhile, it is indicated that the attachment to the fetus can be enhanced by sensitive maternal perception of fetal movements [15-18] and positive child imaging [19-21]. Maternal-fetal attachment is also known to be associated with quickening of the fetus closely [22,23]. Additionally, Shin et al. (2006) showed that more active mother–child interactions during pregnancy caused higher maternal attachment to the fetus [7]. Thus, nursing interventions to let mothers actively interested in the fetus may lead to higher maternal attachment to the fetus.

The abdominal examination method of Leopold’s Maneuvers is a way to determine the position of a fetus inside the uterus by touching the abdomen by hands. This method is expected to enable expectant mothers to perceive the fetal position, and stimulate the awareness of child presence. Hence, we considered that a nursing intervention program using this abdominal palpation method might be useful for the enhancement of maternal-fetal attachment. The aim of this study was to investigate whether a nursing intervention program using abdominal palpation of Leopold’s Maneuvers would improve maternal-fetal relationships of pregnant mothers.

## Methods

### Study setting and participants

The study subjects were singleton pregnant Japanese women under the age of 40 in two women’s hospitals that are under the same management in Shiga, Japan. Because the study started at the 30 weeks of gestation, the participants were recruited from women who were 16 to 28 weeks gestation. They had regular prenatal care at the hospitals, and their pregnancies were progressing normally. The exclusion criteria were pregnant women who were having any complications related to the mother or fetus (for example, low-lying placenta, placenta previa, pregnancy-induced hypertension, intrauterine growth restriction, etc.), pregnancies resulting from advanced assisted reproductive technology, and single mothers. They were recruited from December 2009 through August 2010.

All subjects participated in this study after signing the written consent. This study protocol was approved by the Ethics Committee of Nagoya University School of Medicine.

### Assignment method

**Figure 1:**
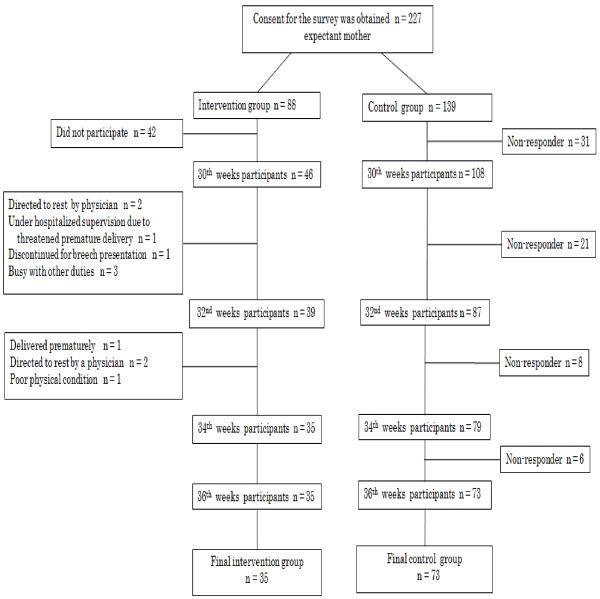
Schematic representation of study’s sampling method.

Of the total of 384 pregnant women in the target weeks’ gestation, 227 (59.1%) agreed to participate in the study. Of those 227 subjects, 88 wished to participate in an intervention program. Thereby, they were non-randomly assigned to the intervention group (n = 88) or the control group (n = 139). In the intervention group, of the initial 88 subjects, 42 did not actually participate in the study at all. Of the 46 participants, seven women participated only once, because two were directed to rest by a physician, one was under hospitalized supervision due to threatened premature delivery, one withdrew due to breech presentation, and three were busy with other duties. Four women participated two times, because one delivered prematurely, two were ordered to rest by a physician due to threatened premature delivery, and one was in poor physical condition. In the end, 35 women participated in the intervention program all three times and completed the questionnaire survey for the 36th week. In the control group of 139 women, 108 completed the questionnaire during the first survey, but 73 during fourth survey. As a result, the final analysis was conducted for 35 (76%) of 46 participants to the first intervention program in the intervention group and 73 (68%) of 108 participants to the first control program in the control group (see Figure 1).

### Procedures

All pregnant women at the hospitals were expected to participate in a pre-mothers’ class to learn about general health care during the pregnancy a total of 3 times at the 30th, 32nd, and 34th weeks’ gestation. Hence, subjects in the intervention group were to participate in both the pre-mothers’ classes and additional intervention programs involving abdominal examinations. Those in the control group were supposed to participate in the pre-mothers’ class (the control program) only. The intervention program (and the control program) was then performed at the 30th, 32nd, and 34th weeks’ gestation.

At the women’s hospitals in this study, all participants regularly received prenatal examinations including ultrasound examinations performed by a physician, and necessary health advice for normal pregnancies from midwives.

### Intervention program

The intervention program was intended to help the pregnant participants to sense the fetal position in the uterus by touching the abdomen by performing an abdominal examination of Leopold’s Maneuvers. Following the pre-mothers’ class (the control program), the nursing intervention program was conducted by three midwives. Midwives explained the fetal position by taking the hand of each participant and touching the buttocks and the back of the fetus together, so that expectant mothers were actually able to touch their abdomens and the large and small body parts of the fetuses. After the practice was completed, a group discussion was held for about 20 min with all of the participants regarding whether they were able to sense the position of the fetus, their thoughts toward the fetus, and so on. Each intervention program took approximately 1 to 1.5 h.

The control group participated in the pre-mothers’ classes. At the study institution, pre-mother classes are held 3 times from the second trimester to the last trimester of pregnancy. The pre-mothers’ classes generally provide information on general health care during pregnancy, such as nutritional intake and daily life, prenatal exercise, preparation for child birth and mental attitude, methods to alleviate delivery labor, infant bathing techniques, and they are also an opportunity to make friends with other participants. Each pre-mothers’ class is held for about 1.5 h. These classes for expectant mothers are common at most of the institutions in Japan.

### Measurements

A self-completed questionnaire survey of the same contents was conducted in the intervention group and the control group at the 30th (baseline), 32nd, 34th, and 36th weeks’ gestation. In the intervention group, subjects completed the questionnaire after practicing the abdominal palpation in the intervention program at the 30th, 32nd, and 34th weeks’ gestation. The survey at the 36th week was done by mail. In the control group, the questionnaire survey was conducted by mail 4 times at the same times as the intervention group. The present study was conducted from November 2009 to December 2010.

The questionnaire covered demographic characteristics, attachment to the fetus, frequency of talking to the fetus, and awareness of the position of the fetus.

For demographic characteristics, the following questions were asked: age, spouse’s age, primigravida or multigravida, nuclear family or extended family, unplanned or planned pregnancy, employed or unemployed, and educational background (to select from junior high-school graduate, high-school graduate, junior college graduate, or graduate of university or higher).

The relationship between the expectant mother and the fetus was examined using the PAI by Muller [24]. We used the Japanese version of the PAI by Tujino [25]. The PAI consists of a 21-item scale that is designed to measure the behavior and feelings of an expectant mother toward the fetus. Each item has 4 Likert-type responses: 4 points are given for “almost always,” 3 points for “often,” 2 points for “sometimes,” and 1 point for “almost never.” The total score ranges between 21 and 84. A higher score is indicative of a higher attachment to the fetus. The Japanese-language version of the PAI has been validated for Japanese women [25]. The Cronbach’s alpha of PAI were 0.89.

The question regarding the frequency of talking to the fetus was as follows: “How many times did you talk to your fetus every day during the last week on average?” The frequency of talking to the fetus is thought to reflect the maternal attachment to the fetus [19-21].

The questionnaire about the awareness of the position of the fetus was intended to measure to what degree expectant mothers perceive the fetus position in the uterus. A 6-item scale with 5 Likert-type responses was prepared by the authors to assess maternal awareness of the fetal position: (1) I perceive that the fetus is moving his/her legs; (2) I perceive that the fetus is moving his/her hands; (3) When I touch the abdomen with my hands, I perceive the position of the back of the fetus; (4) When the fetus moves, I softly touch my abdomen; (5) I imagine the fetus’ character from his/her movement; and (6) I can depict the appearance of the fetus. For each item, 5 points are given for “always,” 4 points for “frequently,” 3 points for “sometimes,” 2 points for “occasionally,” and 1 point for “almost never.” A higher score is expected to show a greater awareness of the fetal position. Internal reliability was examined using Cronbach’s alpha coefficient in this study. This was defined as the fetal position awareness score.

### Statistical analyses

Statistical analyses were performed using chi-square tests or Mann–Whitney *U* tests for categorical variables and Student’s *t* tests for continuous variables. The internal reliability for the questionnaire on PAI and awareness of the fetal position was examined using Cronbach’s alpha coefficient. In order to examine the effects of the intervention, Mann–Whitney *U* tests were employed for comparisons between the intervention group and the control group. For comparison of the time-series data after the intervention, Wilcoxon signed-rank tests were used with a Bonferroni correction because of a 3-time comparison (i.e., of the 30th [baseline] with the 32nd, 34th, and 36th weeks’ gestation). All analyses were 2-sided with *P* values less than 0.05 considered statistically significant. The statistical software package SPSS for Windows J ver. 16.0 (IBM Corporation, Armonk, NY, USA) was used.

## Results

As shown in Table 1, the characteristics of the subjects at baseline showed no significant differences in age, family type, unplanned/planned pregnancy, unemployment, and education between the intervention group and the control group. The proportion of primigravidae in the intervention group tended to be higher, although it was not significantly different between the groups. Prenatal attachment or fetal position awareness also did not differ between primigravidae and multigravidae.

**Table 1:** Characteristics of study subjects (N = 108)

**Characteristics**	**Category**	**Mean (SD) or n (%)**		
		**Intervention group(n = 35)**	**Control group(n = 73)**	***P-value***
Age in years	Expectant mother	30.3 (4.7)	31.8 (4.7)	0.142^a)^
	Spouse	32.5 (6.0)	33.0 (4.9)	0.700^a)^
Parity	Primigravida	26 (74.3%)	42 (57.5%)	0.092^b)^
	Multigravida	9 (25.7%)	31 (42.5%)	
Family type	Nuclear family	28 (80.0%)	58 (79.5%)	0.720^b)^
	Extended family	7 (20.0%)	15 (20.5%)	
Unplanned pregnancy	Yes	14 (40.0%)	31 (42.5%)	0.764^b)^
Employment status	Employed	16 (45.7%)	31 (42.5%)	0.750^b)^
	Unemployed	19 (54.3%)	42 (57.5%)	
Education Level (graduated)	< High school	0 (0%)	3 (4.1%)	0.440^b)^
	High School	7 (20.0%)	13 (17.8%)	
	Specialty school	3 (8.6%)	14 (19.2%)	
	Junior college	10 (28.6%)	21 (28.8%)	
	University or above	15 (42.9%)	22 (30.1%)	

^a) ^Significance determined by t tests between the intervention group and the control group.

^b) ^Significance determined by chi-square tests between the intervention group and the control group.

As for the Cronbach’s alpha coefficient, high internal reliability was verified for the PAI scale (0.884 - 0.926) and the fetal position awareness score (0.740 - 0.854) (see Table 2).

**Table 2:** Internal reliability (cronbach’s α) for PAI score and fetal position awareness score (N = 108)

**Variable**	**Investigation**	**Median (IQR)**	**Cronbach’s α**
PAI ^a)^	30th week	53 (46, 61)	0.884
	32nd week	57 (49, 63)	0.886
	34th week	58 (50, 64)	0.907
	36th week	59 (52, 66)	0.926
Fetal position awareness score	30th week	18 (15, 21)	0.740
	32nd week	20 (16, 23)	0.765
	34th week	21 (17, 24)	0.826
	36th week	22 (18, 25)	0.854

^a)^*PAI*, Prenatal Attachment Inventory score.

At the baseline of the 30th weeks’ gestation, the PAI score and the fetal position awareness score did not differ between the intervention group and the control group. Similarly, the frequency of talking to the fetus was not different between both groups at baseline (see Table 3).

**Table 3:** Changes of maternal-fetal relationship by nursing intervention using abdominal palpations

**Variable**	**Investigation**	**Median (IQR)**		***P-value ***^**b)**^
		**Intervention group (n = 35)**	**Control group (n = 73)**	
PAI ^a)^	30th week	54( 47, 62)	53 ( 45, 61)	0.546
	32nd week	57( 54, 65)**	56( 49, 63)**	0.149
	34th week	61( 55, 70)**	57( 50, 62)*	0.006
	36th week	64( 57, 69)**	58( 51, 63)**	0.021
Frequency of talking to the fetus per day	30th week	3( 2, 6)	3( 2, 5)	0.330
	32nd week	5( 3, 10)**	3( 2, 5)	0.004
	34th week	5( 3, 10)**	4( 2, 6)**	0.005
	36th week	5( 3, 10)**	4( 2, 5)**	0.015
Fetal position awareness score	30th week	19( 16, 22)	18( 15, 21)	0.270
	32nd week	22( 20, 24)**	19( 15, 22)	< 0.001
	34th week	24( 21, 27)**	18( 16, 23)*	< 0.001
	36th week	24( 22, 27)**	21( 17, 23)**	< 0.001

*p < 0.05, **p < 0.01 by the Wilcoxon signed-rank test compared to the 30th week (baseline).

^a)^*PAI*, Prenatal Attachment Inventory score*.*

^b) ^Significance determined by Mann–Whitney U tests between the intervention group and control group.

After the intervention programs, the PAI score in the intervention group was raised significantly at the 32nd, 34th, and 36th weeks’ gestation compared to baseline (*P* < 0.01). Similarly, the control group showed an increase in the PAI scores at the 32nd, 34th, and 36th weeks’ gestation (*P* < 0.05 or *P* < 0.01). Because the score increased more in the intervention group, significant differences between both groups were seen at the 34th and 36th weeks (*P* < 0.01 and *P* < 0.05, respectively).

The frequency of talking to the fetus in the intervention group also increased to be greater at the 32nd, 34th, and 36th weeks’ gestation than at baseline (*P* < 0.01). In the control group, a significant increase was found at the 34th and 36th weeks’ gestation (*P* < 0.01 and *P* < 0.05, respectively). Then, the frequency of talking to the fetus was higher in the intervention group than in the control group at the 32nd, 34th, and 36th weeks (*P* < 0.05 or *P* < 0.01).

Meanwhile, the maternal awareness score of fetal position increased to be significantly greater in the intervention group at the 32nd, 34th, and 36th weeks’ gestation than at baseline (*P* < 0.01), as well as at the 34th and 36th weeks’ gestation in the control group (*P* < 0.05 and *P* < 0.01, respectively). The score in the intervention group was then significantly higher than that of the control group at the 32nd, 34th, and 36th weeks’ gestation (*P* < 0.001) (see Table 3).

## Discussion

In the present study, the PAI score and the frequency to talk to the fetus in the intervention group increased more greatly during pregnancy and were greater after the 32th week or the 34 weeks’ gestation than in the control group. The maternal awareness score of fetal position was also higher in the intervention group after the 32nd weeks’ gestation. Thus, the present nursing intervention using abdomen palpation was shown to be effective for promoting maternal-fetal attachment in normal expectant mothers.

At the 30th weeks’ gestation (the baseline), there were no differences in the PAI score, the frequency of talking to the fetus, the fetal position awareness score, and the social backgrounds between the intervention group and the control group. It is known that the PAI scores are affected by age, marital status, parity, planned pregnancy, and education, and the like [26]. In this study those were not different between both groups, so that both groups under study were considered to have similar social backgrounds.

Pregnant mothers of the normal pregnancy process naturally develop attachment to the fetuses as pregnancy progresses [16,17,27,28]. Gestational age is reportedly the most powerful predictor of maternal-fetal attachment [26]. In this study as well, the maternal attachment to the fetus increased in both groups as pregnancy progressed. However, the PAI scores in the intervention group increased more greatly than in the control group, and the scores of the former subjects were significantly higher after the 34th week of pregnancy than those of the latter. Similarly, the frequency to talk to the fetus was also greater in the intervention group after the 32nd week, which may partly reflect increased maternal-fetal attachment. These findings have demonstrated that the present intervention program improved maternal-fetal attachment.

The present intervention program was designed to facilitate the fetal position awareness of expectant mothers through abdominal examinations of Leopold’s Maneuvers. Earlier studies have suggested that maternal-fetal attachment develops especially after quickening of the fetus [22], and that a mother’s sensitive perception of fetal movements contributes to enhancing attachment to the fetus [15-18], though a recent study did not show the effectiveness of fetal movement counting on attachment [29]. It can be easier to sense fetal movement through the abdominal wall than with the central nervous system [30,31]. In this nursing intervention program, midwives explain the fetal position by taking the hand of each participant and touching the head or the buttocks of the fetus. Such touching of the abdominal wall with midwives to perceive the fetal position could lead to more sensitive perception of fetal activity than fetal movement counting. Actually, the intervention increased the maternal awareness of the fetal position as well as maternal–fetal attachment. These findings suggest that maternal awareness of the fetal position or movements through abdominal palpation could serve to strengthen maternal–fetal attachment. Nursing interventions that enhance fetal position awareness may be an effective method for developing maternal–fetal attachment.

It is also known that maternal attachment toward the child develops during the pregnancy by talking to the fetus or imagining the fetus [32]. Enhanced maternal awareness of the fetal position by abdominal palpation may further promote expectant mothers to feel the fetal presence, imagine the fetus figure, and communicate with the fetus, resulting in the development of maternal attachment to the fetus. In addition, social support is a moderate powerful predictor of maternal-fetal attachment [26]. The present intervention program might have partly worked as social support to pregnant women.

O’Connor et al. (2002) indicated that low socioeconomic status, symptoms of depression, and fewer social resources of expectant mothers and families affect the mother–child relationship [33]. The present subjects were recruited from expectant mothers with normal social backgrounds. The effects of this intervention to high-risk subjects need to be investigated among pregnant women with high-risk backgrounds.

### Study strengths and limitations

The participants were not randomly assigned to the intervention group or the control group. Hence, there may have been a bias to the motivation of the study participants. In addition, the dropout rates after the study started were 23.9% of 11 expectant mothers in the intervention group and 32.4% of 35 expectant mothers in the control group, though the dropouts were due to medical, social, or personal reasons. One of the reasons may be due to a gap of 4 to 6 weeks between the time of obtaining informed consent and that of starting investigation. However, there were no differences in the social backgrounds and maternal–fetal attachment between two groups at baseline.

Maternal-fetal attachment was assessed using a self-reported questionnaire of the PAI. The maternal awareness of the fetal position was measured with an original questionnaire. Their reliability was examined using the Cronbach’s alpha coefficient. They both showed high internal reliability.

The present subjects were 35 women in the intervention group and 73 in the control group, who were recruited in two women’s hospitals that are under the same management. Further RCT studies using more subjects from multiple institutions would be desirable to ascertain the present findings.

## Conclusions

The present findings have suggested that nursing intervention using abdominal palpations of Leopold’s Maneuvers can enhance maternal awareness of fetal positions, maternal–fetal attachment and the frequency of the mother talking to the fetus. Further random controlled trials are warranted to ascertain this observation.

## Competing interests

The authors declare that they have no competing interests.

## Authors’ contributions

Both authors were responsible for designing, data processing, statistical analysis, interpretation and writing up the final article and gave the final approval of the manuscript to be published.
